# Interventional radiology management of disc herniation: a narrative review

**DOI:** 10.1007/s11547-026-02236-7

**Published:** 2026-06-15

**Authors:** Salvatore Alessio Angileri, Carolina Lanza, Serena Carriero, Marianna Platì, Chiara Perazzo, Maria Francesca Girlando, Donatello Berloco, Maria Luisa Franzone, Chloe Yeabin Jung, Giovanni Maria Rodà, Pasquale Grillo, Pierluca Torcia, Pierpaolo Biondetti, Anna Maria Ierardi, Gianpaolo Carrafiello

**Affiliations:** 1 Department of Diagnostic and Interventional Radiology, Foundation IRCCS Ca’ Granda Ospedale Maggiore Policlinico, Milan, Italy; 2 Postgraduate School of Diagnostic and Interventional Radiology, University of Milan, Milan, Italy; 3 Università Degli Studi Di Milano, Via Festa del Perdono 7, Milan, Italy

**Keywords:** Disc herniation, Spine procedure, Decompression, Pain management, Minimally invasive procedure, Interventional radiology

## Abstract

Disc herniation (DH) is a common cause of spinal pain and radiculopathy. While most patients improve with conservative treatment, cases with persistent symptoms may require further intervention. In this context, interventional radiology (IR) offers image-guided, percutaneous techniques as a minimally invasive therapeutic option in the stepwise management of DH. This narrative review, conducted in accordance with SANRA principles, analyses the diagnostic and therapeutic role of IR in cervical, thoracic, and lumbar DH. A structured literature search was performed in PubMed/MEDLINE and Scopus to identify studies evaluating mechanical, thermal, chemical, and regenerative approaches. Reported outcomes included pain relief, functional recovery, complication rates, and need for reintervention. Evidence suggests that percutaneous intradiscal procedures may achieve meaningful short- and medium-term clinical improvement in carefully selected patients with contained herniations and clinicoradiological concordance. These techniques are associated with reduced invasiveness, shorter recovery times, and lower complication rates when performed by experienced practitioners. Imaging plays a central role in diagnosis, patient selection, procedural planning, as well as technical accuracy and safety. Nonetheless, current evidence remains heterogeneous and largely based on observational studies, with limited long-term comparative data. Further multicentre prospective trials and standardized outcome reporting are required to better define indications and lasting effectiveness.

## Introduction

Interventional radiology (IR) plays a key role in the management of disc herniation (DH), offering increasingly effective and safe minimally invasive treatment options [1].

The management of DH generally follows a multistep approach: most patients achieve symptom relief with conservative therapies [2], but a small percentage require additional interventions [3]. Within this context, IR procedures constitute a broad, continuously evolving spectrum of interventions, encompassing chemical, thermal, and mechanical ablation techniques, as well as cell-based therapies (e.g. platelet-rich plasma, mesenchymal stem cells) and biomaterial implantation (e.g. hydrogel) [4].

Compared with traditional surgery, these percutaneous interventions allow targeted treatment of disc pathology while limiting surgical trauma, infection risk, postoperative adhesions, and other complications associated with open approaches [5, 6]. In addition, they are typically performed under local anaesthesia, on an outpatient or day-hospital basis, enabling shorter recovery times and faster return to daily activities [7].

Despite their growing availability, the indications, advantages, and safety profiles of these IR techniques remain heterogeneous in the literature. This narrative review aims to provide an overview of the evolving diagnostic and therapeutic role of IR in managing DH, with a focus on its technical diversity, clinical outcomes, advantages, complications, and ongoing limitations. By synthesizing current evidence, we aim to explore the future potential in the field of minimally invasive spine care and to clarify the position of IR within the multidisciplinary management of DH.

## Materials and methods

A structured literature search was conducted in accordance with the Scale for the Assessment of Narrative Review Articles (SANRA) framework to synthesize current knowledge. This search was performed in PubMed/MEDLINE and Scopus to identify articles published up to 30 September 2025, using combinations of keywords including “disc herniation”, “discogenic pain”, and “percutaneous disc decompression”. The reference lists of relevant articles were meticulously reviewed to identify additional studies.

Eligible publications included randomized controlled trials, prospective or retrospective observational studies, comparative studies, systematic reviews, and meta-analyses evaluating percutaneous image-guided treatments for cervical, thoracic, and lumbar DH in patients with persistent symptoms despite conservative therapy, with reported outcomes including pain relief, functional improvement, reintervention rates, or complications. Case reports, conference abstracts, non-peer-reviewed publications, non-English articles, and studies lacking sufficient methodological detail were excluded. After the removal of duplicates, titles and abstracts were screened for relevance, followed by full-text review of potentially eligible articles; data on technique, outcomes, complications, and follow-up were then extracted and synthesized qualitatively. For clarity and consistency, evidence was grouped into mechanical, thermal, chemical, and regenerative intervention categories, and differences in methodology and patient selection were considered when interpreting variability in reported outcomes.

This review is exclusively based on previously published literature and did not involve direct patient recruitment, use of individual patient data, experimental interventions, or animal studies; therefore, approval from an Institutional Review Board or Ethics Committee and informed consent were not required.

## Definitions and physiopathology

In 2014, Fardon et al. [8] proposed a new definition and classification of DH as a “localized or focal displacement (< 25%) of disc material beyond the limits of the intervertebral disc space”, discriminating it from disc bulging [9].

DH can be classified as contained or uncontained [8]. A contained hernia is covered by the posterior longitudinal ligament and/or outer annulus fibres, whereas an uncontained hernia lacks such covering [8, 9].

The morphology of the displaced material provides an additional classification criterion. When the herniated disc material is smaller than the disc base, it is defined as a protrusion [8, 9]. In contrast, an extrusion refers to a herniation in which a small base supports a larger disc fragment displaced into the spinal canal; extrusion hernias are usually uncontained [8, 9]. Extrusions can be further subclassified as sequestration or migration. Sequestration occurs when the extruded fragment loses continuity with the disc of origin, while migration refers to displacement of the disc fragment from its original position, regardless of continuity [8, 9].

DH can also be categorized by the anatomical location of herniation on the axial plane as central, posterolateral, intra-foraminal, or extra-foraminal; this classification is clinically relevant, as the hernia’s location correlates with specific symptom patterns [8].

DH may involve any intervertebral segment, although the lumbar spine is most commonly affected. Specifically, the L4-L5 and L5-S1 segments account for the majority of symptomatic cases, often resulting in low back pain and sciatica [10]. The cervical spine is also frequently affected, most commonly at C5-C6 and C6-C7, resulting in cervical radiculopathy, characterized by neck pain radiating to the arm, sensory disturbances, and motor weakness in the corresponding myotome [11, 12].

The pathophysiology of DH is multifactorial and cannot be explained solely by mechanical compression. Simple nerve root compression predominantly causes paresthesias or sensory deficits and is rarely sufficient to generate significant pain [13]. This implies that radicular pain involves both biochemical and neurogenic pathways. Numerous studies have further demonstrated that pro-inflammatory mediators, such as prostaglandins, tumour necrosis factor *α* (TNF-*α*), and interleukins (IL-1*β*, IL-6), are highly expressed in degenerative discs [14, 15]. Released due to disc degeneration and migration of nucleus pulposus (NP) material beyond the annulus fibrosus, these molecules trigger local inflammatory response and increase the sensitivity of surrounding nociceptive nerve terminals [15, 16].

Additionally, increased innervation within degenerated discs contributes to pain. Histological studies have documented a higher density of nerve fibres immunoreactive for substance P, a neuropeptide involved in pain signalling, in severely degenerated discs [17, 18]. Normally absent in healthy discs, these fibres can penetrate deeply into the disc stroma following degenerative and inflammatory processes [10, 18].

Overall, DH pathogenesis results from a multifaceted interplay of mechanical factors [13, 19], local inflammatory processes [14, 15], and neuroplastic remodelling [17, 18], ultimately leading to activation and persistence of nociceptive responses.

## Imaging tools

Imaging plays a key role in the diagnosis of DH by providing information on disc morphology, nerve involvement, and potential differential causes of symptoms [20, 21]. However, imaging is not recommended during the first six weeks of symptoms unless severe neurological deficits, suspected tumours or infections, or cauda equina syndrome are present [20, 21].

Standard radiographs (X-rays) remain a quick and inexpensive method for assessing spinal alignment and degenerative changes, although they provide only indirect evidence of DH [22, 23]. Computed tomography (CT) offers superior visualization of bony structures and calcifications but has limited soft-tissue contrast [24, 25]. CT myelography is generally reserved for cases where magnetic resonance imaging (MRI) is contraindicated or unavailable [24, 26].

MRI is considered the gold standard for DH diagnosis [27, 28], as it allows direct visualization of intervertebral discs, nerve roots, and surrounding soft tissues with an accuracy exceeding 90%, without exposure to ionizing radiation [[27]. Gadolinium-enhanced MRI is typically reserved for suspected infection or neoplastic processes [29]. In addition, MRI can demonstrate disc dehydration, vertebral endplate changes, and inflammatory signs that are valuable for treatment planning.

Electrodiagnostic tests, such as electromyography and nerve conduction studies, complement imaging by characterizing nerve injury and quantifying its severity [30]. Overall, MRI remains the primary diagnostic modality, while CT and electrophysiological testing serve as adjuncts when clinically indicated.

### MRI protocol

The standard MRI protocol for DH includes sagittal and axial T1- and T2-weighted sequences, sometimes combined with fat suppression or short tau inversion recovery (STIR) imaging. Sagittal T1-weighted images are used to assess vertebral alignment and disc morphology, whereas sagittal T2-weighted sequences highlight disc dehydration and neural compression. Axial T2-weighted images are essential for identifying the location and extent of DH, while STIR sequences are useful for detecting inflammation or oedema. Axial T1-weighted sequences are optional but may provide additional information by demonstrating epidural fat displacement and foraminal narrowing [27, 29]. Table 1 summarizes the main MRI sequences used for DH assessment, including the corresponding imaging planes, key features, and the specific diagnostic information provided by each sequence.

**Table 1:** Standard MRI protocol for DH evaluation: sequences, imaging planes, and key imaging features

MRI sequence	Imaging plane	Key features	Imaging details provided
T1-weighted	Sagittal	Anatomical overview, bone marrow evaluation	Vertebral alignment, bone marrow changes, disc contour, epidural fat
T2-weighted	Sagittal	Assessment of hydration and nerve compression	CSF-bright contrast, disc degeneration, herniation size and effect on dural sac
T2-weighted	Axial	Localization and extent of herniation	Central/paracentral/foraminal location, nerve root contact or compression
T1-weighted (optional)	Axial	Foraminal anatomy, epidural fat displacement	Precise foraminal narrowing, disc–fat interface
Fat-suppressed/STIR	Sagittal/Axial	Highlight inflammatory or oedematous changes	Bone marrow oedema, soft-tissue inflammation

MRI also evaluates changes in the signal of the bone marrow and vertebral plate according to Modic’s classification [31]: type I (oedema), type II (fatty alteration), and type III (sclerosis). These changes have been associated with pain severity and clinical outcomes [32, 33].

Furthermore, emerging artificial intelligence (AI) tools enable automated detection [34] and classification [35] of DH, thereby improving diagnostic reliability and may improve diagnostic accuracy and workflow efficiency [36]. However, most AI models are derived from retrospective, single-centre data sets, limiting their generalizability. Heterogeneity in imaging protocols, scanner vendors, and annotation standards further constrains clinical translation. At present, AI systems serve primarily as decision-support tools and continue to require expert radiological oversight. Prospective, multicentre validation studies are essential prior to routine clinical implementation.

## Treatment

### Conservative therapies

**Fig. 1:**
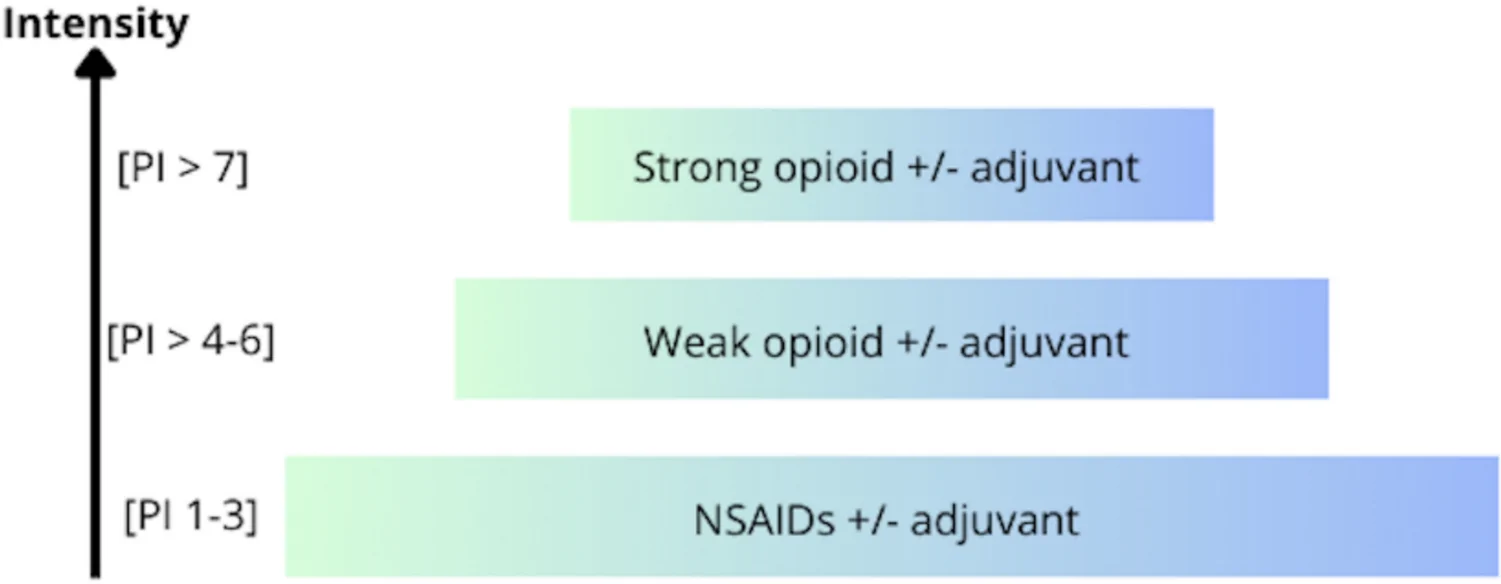
Conservative pain management strategy for DH based on pain intensity [42]

Initial management of symptomatic DH typically relies on conservative treatment to achieve pain relief, facilitate functional recovery, and allow for the potential spontaneous regression of herniated disc material [37]. This approach generally includes rest, activity modification, physical therapy, and short-term use of analgesic or anti-inflammatory medications over a 4- to 6-week period, with epidural steroid injections reserved for selected patients with persistent symptoms [38, 39]. Despite their efficacy in numerous cases, conservative therapies are often limited by incomplete pain alleviation and medication-related adverse effects [40]. Consequently, patients who fail to show improvement after an adequate course of conservative treatment require further diagnostic evaluation, including appropriate imaging, to confirm disc pathology, establish clinicoradiological concordance, and exclude alternative conditions that may mimic DH [25, 37, 41]. Based on these findings, image-guided percutaneous or surgical interventions may then be considered. Figure 1 illustrates the stepwise conservative pain management strategy for DH based on pain intensity.

### Percutaneous therapies

Percutaneous treatments are indicated in selected patients with disc-related symptoms that are unresponsive to conservative therapies [43, 44].

In the current literature, percutaneous procedures are commonly classified into two main categories based on their primary therapeutic target and mechanism of action [45, 46].

The first category comprises periradicular procedures, including epidural steroid injections and periradicular radiofrequency ablation. The primary objective of these procedures is to modulate inflammation and nociceptive transmission at the nerve root level, thereby providing symptomatic relief in patients experiencing predominantly radicular pain [38, 47]. These techniques do not directly modify disc morphology and are therefore primarily indicated for inflammatory or irritative radiculopathy.

The second category comprises intradiscal procedures that directly target the intervertebral disc to reduce intradiscal pressure, decrease herniation volume, and, in selected cases, promote tissue repair. This group encompasses mechanical [43], thermal [48], chemical [49], and biomaterial-based approaches [50, 51] and is primarily intended for patients with mechanically driven discogenic pain and contained DHs.

Across studies, the ideal candidates for percutaneous intradiscal treatments are patients with symptomatic, contained DH occupying less than one-third to one-half of the spinal canal on MRI, with preserved disc height, absence of significant spinal instability, and clear clinicoradiological concordance. Absolute contraindications include cauda equina syndrome, sphincter dysfunction, progressive neurological deficit requiring urgent surgery, sequestered disc fragments, infection, spinal instability, and uncorrected coagulation disorders [50, 52].

#### Percutaneous techniques of disc decompression

The main percutaneous disc decompression techniques, together with their mechanisms of action and principal advantages, are summarized in Table 2. Although these approaches differ technically, their shared objective is to reduce intradiscal pressure and mechanical nerve root compression while preserving disc structure.

**Table 2:** Categories, mechanisms, advantages, and limitations of percutaneous treatments for DH

Category	Mechanism of action	Advantages	Limitations
Mechanical decompression	Nucleotome: Partial removal of the nucleus pulposus reduces intradiscal pressure and nerve root compression	Preservation of disc anatomy	Limited effectiveness in large or migrated herniations; risk of incomplete decompression and recurrence
	Herniotome: Mechanical extraction of the herniated fragment or part of the protrusion through a percutaneous trocar	Reduced risk of fibrosis	Not suitable for calcified or sequestered herniations
Thermal decompression	Percutaneous laser disc decompression (PLDD): Laser-induced vaporization of the nucleus pulposus, resulting in intradiscal pressure reduction	Minimally invasive; preservation of spinal stability; low complication rates	Risk of thermal injury or nerve damage if used improperly; limited effectiveness in large herniations; high equipment cost
	Coblation: Ionic plasma-mediated ablation of nuclear tissue at low temperature (40–70 °C), resulting in volume and pressure reduction	Low risk of thermal injury; effective symptom relief; biochemical modulation	Requirement for hydrated disc tissue; risk of incomplete decompression and recurrence
Chemical decompression	DiscoGel®: Ethanol-induced dehydration and necrosis of nucleus tissue, along with pressure reduction and neurolytic effects	Effectiveness in contained herniations; the only technique used in instability	Risk of leakage and chemical radiculitis; contraindicated in large extrusions; requirement for strict dose control
	O_2_-O_3_ Therapy: Oxidative degradation of nucleus components with associated intradiscal volume reduction and anti-inflammatory effects	Anti-inflammatory and analgesic effects; rapid recovery	Short-term efficacy; standardization of variable ozone concentrations; requirement for multiple treatment sessions
Biomaterial/regenerative therapies	Mesenchymal stem cell (MSC): Differentiation into nucleus pulposus-like cells and paracrine release of trophic factors promoting anabolic repair of disc tissue	Regenerative potential; potential restoration of disc function; low immunogenicity	Limited long-term data; high cost; technical complexity; risk of uncontrolled differentiation
	Plasma-rich plasma (PRP): Release of growth factors promoting matrix synthesis, cell proliferation, and anti-inflammatory effects	Autologous and safe; easy preparation; anti-inflammatory and trophic effects	Variability in composition; limited regenerative depth; transient therapeutic effect requiring repeated injections
	Hyaluronic acid (HA): Restoration of nucleus matrix hydration and viscoelasticity with support of cellular adhesion and regeneration	Biocompatible; improved elasticity and shock absorption; promotion of local healing	Temporary effect; lack of structural defect correction; limited evidence for long-term benefit

##### Percutaneous accesses

**Fig. 2:**
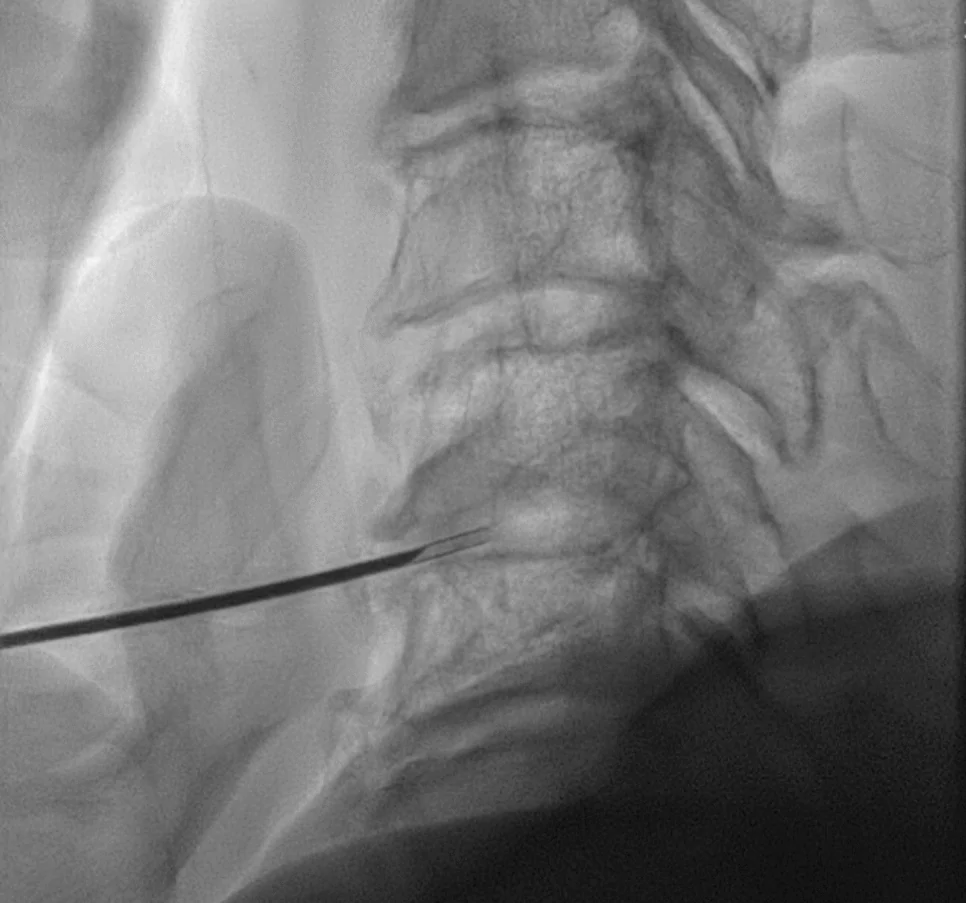
Fluoroscopic guidance for percutaneous cervical disc access

**Fig. 3:**
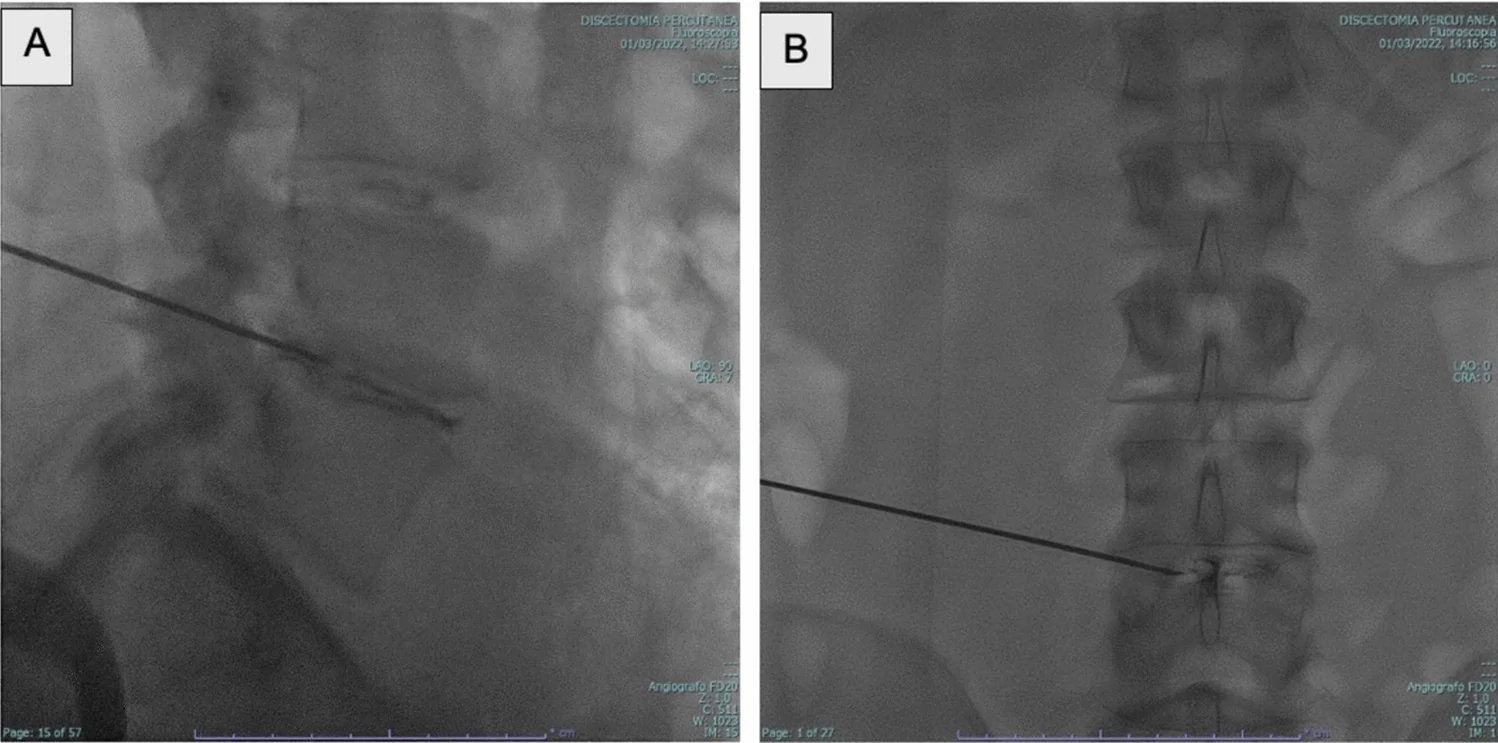
Fluoroscopic guidance during percutaneous lumbar access. **A** Lateral view showing correct needle positioning within the intervertebral disc space via a posterolateral approach, with discography using contrast agent. **B** Anteroposterior view confirming midline orientation and adequate depth prior to decompression

Percutaneous access varies according to spinal level, herniation type and location, and operator expertise [52, 53]. Procedures are performed under fluoroscopic guidance, though CT or MRI may also be used [50, 54]. Figure 2 illustrates cervical access, while Fig. 3 depicts lumbar access; in both cases, fluoroscopy is used to confirm correct trajectory and depth prior to the therapeutic phase.

For cervical levels, an anterolateral approach is employed: the neck is extended, and the carotid sheath is gently retracted laterally to protect vascular structures, while the trocar is advanced between the oesophagus and the carotid artery [53, 55].

In the thoracic region, a posterolateral approach is preferred, aligning the fluoroscope along the disc plane with a slight oblique angle to safely reach the target [56].

For lumbar DHs, a posterolateral approach is also favoured, with the needle inserted obliquely to reach the annulus just anterior to the superior articular process. At the L5-S1 level, a steeper trajectory may be necessary due to the iliac crest or spinal curvature [4, 55].

After confirming the correct placement within the NP, the therapeutic procedure—mechanical, chemical, or thermal—is performed. Table 3 provides an overview of percutaneous spinal access techniques by treated spinal level and imaging guidance.

**Table 3:** Fluoroscopic spinal access techniques and needle specifications across different spinal levels

Spinal level	Access type	Imaging guidance/orientation	Skin entry point	Needle type/gauge	Technical notes/considerations
Cervical	Anterolateral	Fluoroscopic, with direct palpation guidance	Between the carotid-jugular bundle laterally and the oesophagus medially	19G, 80 mm	Displace carotid and jugular laterally using two fingers; puncture the disc between them
Thoracic	Posterolateral	Fluoroscopic, 35° lateral rotation from AP and craniocaudal tilt aligned with the disc plane	Along the X-ray beam axis, between the “double rings” (rib head and pedicle)	17G, 160 mm	Puncture between the two ring-shaped shadows to reach the disc safely and obtain optimal projection
Lumbar	Posterolateral	Fluoroscopic, approximately 45° beam rotation (“Scottie dog” view)	Approximately 10 cm from the spinous process of the superior vertebra	17G, 160 mm	Advance the needle anterior to the superior articular process (“Scottie dog” projection); in AP view, stay lateral to dural sac; in LL view, avoid retroperitoneum
Lumbar (L5-S1)	Posterolateral	Fluoroscopic, 45° lateral oblique projection	Approximately 10 cm from the spinous process, just above the iliac crest	17G, 160 mm (curved needle if needed)	Skin entry point slightly more cranial than L4-L5; verify AP and LL projections; advance with rotational AP movements until anterior vertebral margin

##### Mechanical decompression

Disc decompression using mechanical techniques is based on partial removal of the NP, typically 1–3 g [57]. The principal objective of these techniques is to reduce intradiscal pressure exerted on the nerve root [57].

A review of the present literature on percutaneous procedures reveals considerable variability in reported outcomes. This heterogeneity is largely attributable to study designs, as many investigations were conducted with relatively small sample sizes. Consequently, these studies often had limited statistical power, impeding their ability to detect statistically significant differences between techniques, even in the presence of clinically meaningful improvements.

Several studies have demonstrated that percutaneous procedures are associated with improvements in terms of pain reduction and mobility compared with conservative therapy, with benefits observed not only in the short term but also at 12–24 months [52]. In contrast, another study yielded no statistically significant differences among various percutaneous decompression techniques, including mechanical decompression and radiofrequency decompression [58]. These techniques demonstrated comparable outcomes with respect to Numeric Rating Scale (NRS) and Oswestry Disability Index (ODI) scores, hospital stay duration, and return-to-work rates [58]. Similarly, multiple studies have found no statistically significant differences in effectiveness among open discectomy, microdiscectomy, and percutaneous decompression techniques [59] [60].

In long-term evaluations, some studies have reported marginally lower patient satisfaction following mechanical percutaneous procedures compared with surgical interventions [59]. This finding is noteworthy despite the lower complication rates, shorter hospital stays, and reduced healthcare costs associated with mechanical percutaneous techniques [59]. Although infrequent, potential complications of these techniques include discitis and, in rare cases, cauda equina syndrome [61, 62].

##### Thermal decompression

Percutaneous decompression using thermal techniques is predicated on the utilization of devices, such as lasers, that generate heat capable of inducing the controlled vaporization of a portion of the NP [54, 63]. The decompressive effect is associated not only with a reduction in disc volume, but also with physical and biochemical alterations in the intervertebral disc [54, 63].

The effectiveness of these techniques largely depends on disc features and is limited when dehydration is severe, as reduced water content impairs the thermal mechanism of action.

A review of the available literature suggests that percutaneous thermal procedures can lead to a significant reduction in pain and improvements in quality of life, with superior results compared with epidural steroid injections [54]. Furthermore, it is documented that a therapeutic strategy involving percutaneous thermal decompression, followed by surgical intervention in cases showing minimal to no response to other treatments, can produce outcomes comparable to those of a primary microdiscectomy approach. [64]. This finding lends further credence to the role of thermal techniques as a viable intermediate option within a gradual, stepwise therapeutic pathway [64].

Complications associated with thermal techniques are related to the use of heat. Among the most frequently described are discitis [65, 66], nerve root irritation or damage [62, 67], which can manifest as pain or transient neurological deficits, and a high incidence of pain at the needle insertion site. These symptoms are generally transient and tend to resolve within two weeks of the procedure. As documented in the existing literature, rare complications, including vertebral osteonecrosis and cauda equina syndrome, have been associated with technical errors [62]. This underscores the paramount importance of meticulous patient selection and rigorous procedural execution.

##### Chemical decompression

Percutaneous decompression employing chemical techniques is predicated on the intradiscal administration of agents that reduce the volume of the NP through chemical mechanisms [55]. The most prevalent methods in this field include the injection of gelified ethanol (DiscoGel®), a technique that induces controlled dehydration of the NP [55], and the use of ozone, which exploits its oxidative properties to degrade disc tissue [57]. The efficacy of these methods depends on the integrity of the annulus fibrosus, thereby rendering them applicable primarily to cases of contained DH [68].

Data pertaining to the clinical outcomes of chemical decompression are derived primarily from observational studies and non-randomized comparative analyses [49, 68]. A review of the available literature indicates that DiscoGel may lead to a significant reduction in pain and functional improvement in patients with contained DH, with benefits maintained even at medium- and long-term follow-up [69]. Similar results were reported in a recent retrospective analysis of patients with cervical DH treated with DiscoGel, which demonstrated a significant reduction in pain intensity in selected cases, thereby suggesting a potential expansion of its indication to the cervical spine [70].

Despite the favourable safety profile of this technique, there have been documented reports of complications in the existing literature. These include cases of discitis, leakage of injected material leading to radicular irritation, transient inflammatory reactions, and, in exceedingly rare instances, anaphylactic reactions [69].

##### Biomaterial implantation

The utilization of biological materials in the treatment of DH constitutes a novel frontier, with the objective of restoring disc homeostasis rather than decreasing its volume [55]. The primary aim is to modulate inflammatory processes, stimulate extracellular matrix synthesis, and enhance the biochemical properties of the disc [51, 71]. Despite the sound underlying biological rationale and initial experimental findings suggesting anti-inflammatory effects and potential matrix support, clinical evidence remains scarce [71]. Existing published studies are characterized by heterogeneous protocols, modest patient sample sizes, and follow-up periods that are inadequate for reliably evaluating long-term outcomes [51]. Consequently, it remains premature to draw definitive conclusions regarding structural disc restoration or the recovery of biomechanical properties over time.

Further concerns pertain to the potential risks associated with these therapeutic interventions, including infection, adverse immune responses, and biomaterial migration. These risks affirm the necessity for prolonged clinical monitoring and cautious interpretation of early results. In the light of these considerations, the utilization of biological materials in the treatment of DH should currently be regarded as an experimental intervention rather than a proven therapeutic modality. This perspective is contingent upon the conduct of randomized, long-term clinical trials designed to meticulously delineate their efficacy, safety profile, and specific indications.

### Thoracic disc herniation: an underexplored region

Thoracic DH is an unusual clinical entity, accounting for only 0.25–1% of all DHs [72]; consequently, it remains relatively underexplored in the clinical literature. The T11-T12 segment is the most frequently affected site in the thoracic region [73].

In clinical practice, thoracic DH is characterized by a higher prevalence of myelopathy compared to cervical and lumbar levels [72, 73]. The presence of spinal cord compression, progressive neurological deficit, or intramedullary signal changes on MRI typically indicates the necessity for surgical decompression [72, 74].

Image-guided percutaneous procedures are recommended only for patients without myelopathy and with predominantly radicular symptoms [72, 73]. However, the limited epidural space, the common central location of thoracic herniations, and a higher incidence of calcified discs increase the technical complexity and potential neurological risks [72, 73].

The current evidence supporting minimally invasive or percutaneous approaches in the thoracic spine is primarily derived from small case series and technical reports, including laser and endoscopic procedures [56, 75, 76]. Consequently, these techniques should currently be regarded as complementary options in carefully selected cases rather than established alternatives to surgery [72, 74].

### Surgical treatments

Surgical intervention is indicated for ongoing or intense radicular pain, progressive neurological deficit, or cauda equina syndrome. In the absence of these conditions, guidelines generally recommend surgery only after 6–12 weeks of unsuccessful conservative treatment or in cases of neurological impairment [5, 6]. Evidence indicates that surgery may expedite symptom relief in the short term, although long-term outcomes are often comparable to delayed intervention [41].

For lumbar DH, microdiscectomy remains the gold standard, with its well-established efficacy and safety [77, 78]. In the cervical spine, anterior cervical discectomy and fusion (ACDF) is most commonly performed [79, 80], while cervical disc arthroplasty represents a valid alternative in appropriately selected patients [79].

Conversely, in thoracic DH, minimally invasive techniques have shown effectiveness, whereas conventional surgery is typically reserved for cases in which conservative and interventional treatments have been exhausted [74, 76].

Table 4 provides a summary of a practical clinical decision-making algorithm integrating conservative, percutaneous, and surgical management strategies for DH.

**Table 4:**
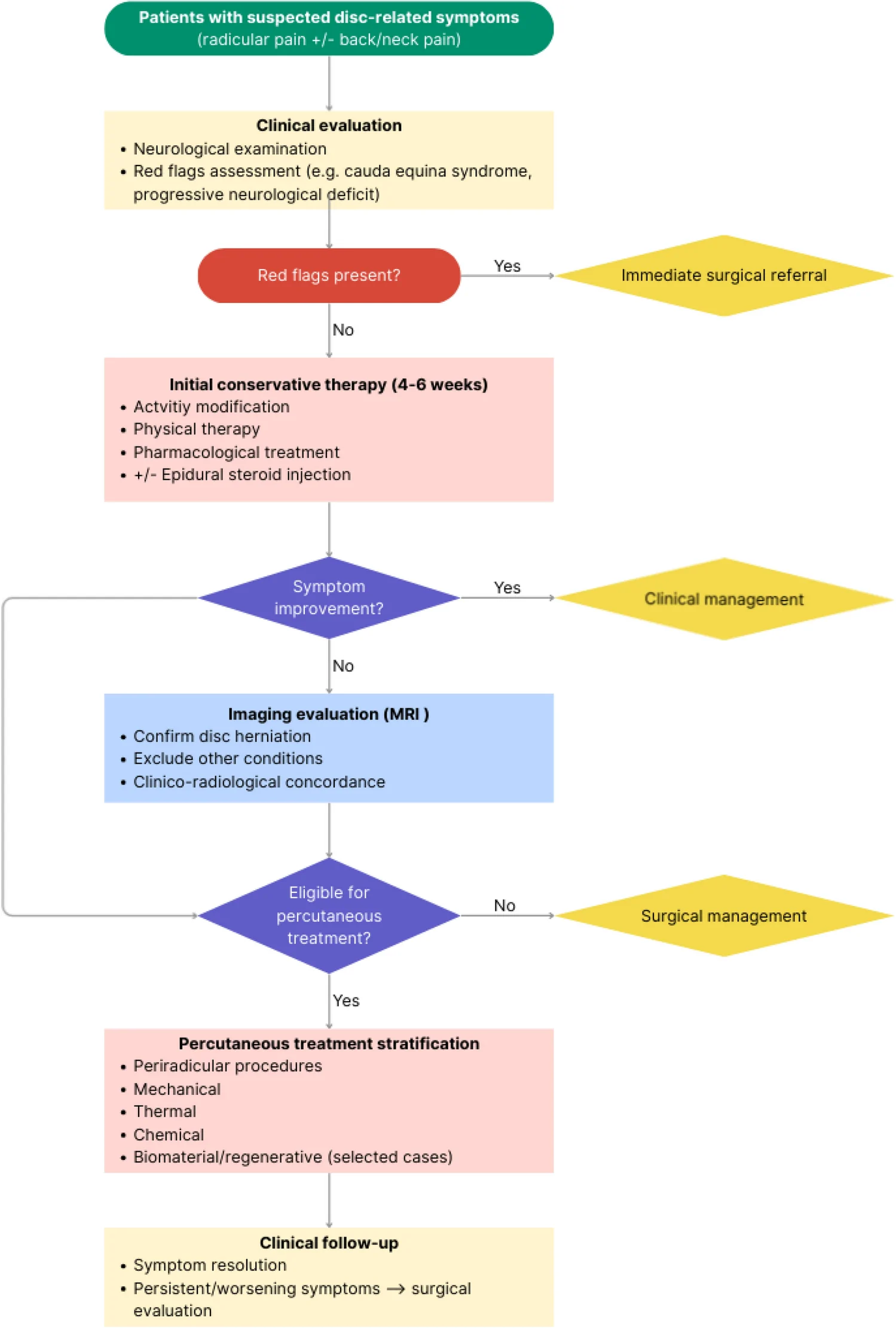
Clinical decision-making algorithm for the management of patients with suspected DH

## Discussion

In recent years, there has been a substantial increase in the development of percutaneous techniques for the treatment of symptomatic DH [81]. This trend has emerged in response to the high prevalence of cervical and lumbar disc disease and the need for less invasive treatment alternatives for patients who do not require urgent surgical intervention [3, 57]. IR plays a key role in this advancement, applying advanced diagnostic imaging with targeted percutaneous techniques to address selected disc conditions with lower invasiveness and expedited recovery compared with conventional open procedures [38, 57].

From an epidemiological perspective, cervical and lumbar DHs represent the majority of symptomatic cases, particularly at the C5-C6, C6-C7, L4-L5, and L5-S1 levels [11, 27]. Thoracic DH, by contrast, remains uncommon and under-researched, primarily due to technical challenges associated with regional anatomy and the elevated prevalence of central or calcified herniations, which limit the feasibility of percutaneous techniques [72, 73]. This contributes to the reduced standardization of IR approaches in the thoracic spine relative to the cervical and lumbar regions [75, 82].

Imaging plays a fundamental role in both diagnosis and treatment. MRI is the gold standard for DH diagnosis, thanks to its high spatial resolution and excellent visualization of soft tissues, nerve roots, and disc morphology without exposing patients to ionizing radiation [24, 25]. However, CT and fluoroscopy remain indispensable in planning and performing percutaneous procedures, ensuring targeting accuracy and procedural safety [25].

Conservative management, which typically involves a 4- to 6-week course, remains the primary approach due to the potential for spontaneous herniation regression [37]. In cases where symptoms persist, imaging-guided percutaneous procedures may serve as a suitable alternative to surgery, especially for contained hernias, clinicoradiological concordance, absence of progressive neurological deficits or signs of surgical urgency, and no segmental instability or sequestered or migrated fragments [57]. In this context, IR presents as a viable therapeutic option, mainly aimed at pain control and functional recovery, with benefits of reduced invasiveness and faster healing, offering clear advantages over traditional therapies [38, 52].

Percutaneous intradiscal techniques encompass a diverse array of approaches, including mechanical, thermal, and chemical methods, as well as more recent developments in biomaterial and regenerative treatments [43, 46, 57]. Despite their diversity, these techniques share a common rationale: to mitigate intradiscal pressure or to modulate the inflammatory environment. However, they exhibit notable distinctions in their respective mechanisms of action, anatomical indications, and the robustness of supporting scientific evidence. Current literature review suggests that, in appropriately selected patients, these procedures have the potential to elicit a substantial improvement in pain and disability, with short- and medium-term outcomes comparable to those of surgical interventions [[52, 58]. Nevertheless, it is imperative to note that the heterogeneity of available studies, including small sample sizes, variable inclusion criteria, and inconsistent follow-up, limits the interpretability of these results, highlighting the importance of careful patient selection [57].

Comparison with surgery should be seen as complementary rather than competitive. Surgery continues to play a pivotal role in cases of progressive neurological deficit, cauda equina syndrome, or anatomical conditions unsuitable for percutaneous approaches [5, 6, 41]. IR techniques constitute valid options that serve as a bridge between conservative therapy and surgery, providing an effective, minimally invasive option for patients who do not respond to medical management and do not meet criteria for urgent surgical intervention [41, 58].

IR techniques are generally associated with a low incidence of complications, which the extant literature characterizes as infrequent [8, 57]. It has been established that the most concerning potential complication is spondylodiscitis, although its occurrence is rare [8, 57]. The remaining documented complications are mostly related to technical errors or inappropriate patient selection [62]. This points out the critical importance of accurate clinical and radiological assessment, along with a high level of technical expertise on the part of the operator, in order to ensure procedural safety and effectiveness [62].

Biomaterial and regenerative therapies represent a promising frontier, potentially shifting the paradigm from simple decompression to biological modulation of the disc [71, 83]. While early results are encouraging, there is currently insufficient clinical evidence to support routine use, and randomized trials with long-term follow-up are needed.

This review is limited by its narrative design and the absence of formal risk-of-bias assessment or quantitative grading of evidence. Although a structured literature search was performed, the included studies may not fully represent the entire body of available evidence, and heterogeneity in study design and outcome reporting limits direct comparisons across techniques. Furthermore, there is a notable lack of multicentre prospective trials directly comparing percutaneous and surgical techniques, particularly regarding long-term clinical and functional outcomes, which restricts the strength of conclusions that can be drawn about their relative effectiveness.

In conclusion, IR techniques are a valid approach for managing DH in selected patients. They offer clinical outcomes comparable to traditional surgery in terms of pain control and functional recovery while being less invasive and having reduced impact on surrounding tissues. They are also more cost-effective. Advanced imaging techniques facilitate accurate instrument placement, thereby improving procedural safety and overall treatment effectiveness.
